# Development of quality indicators of transfer and transition in adolescents and young adults with congenital heart disease

**DOI:** 10.1186/s12913-023-10183-6

**Published:** 2023-10-25

**Authors:** Corina Thomet, Philip Moons, Markus Schwerzmann, Fabienne Schwitz

**Affiliations:** 1 Center of Congenital Heart Disease, Department of Cardiology, Inselspital, Bern University Hospital, University of Bern, Bern, 3010 Switzerland; 2 Department of Public Health and Primary Care, KU Leuven, University of Leuven, Leuven, Belgium; 3 Institute of Health and Care Science, University of Gothenburg, Gothenburg, Sweden; 4 Department of Paediatrics and Child Health, University of Cape Town, Cape Town, South Africa

**Keywords:** Heart defects, Congenital, Cardiac care facilities, Transition to adult care, Quality indicators, Health care, Europe

## Abstract

**Background:**

Quality indicators are crucial in evaluating and comparing the quality of healthcare services. In the case of congenital heart disease, transition programmes for adolescents have been recommended to ensure uninterrupted healthcare and lifelong care. It is necessary to establish quality indicators in order to facilitate the evaluation of programme quality and to allow comparison between different centres. The objective of this study is therefore to develop a set of quality indicators for the transition of adolescents with congenital heart disease.

**Methods:**

The RAND/UCLA appropriateness method was employed in a four-step process to develop a set of quality indicators. First, a literature search was conducted on the dimensions of transitional care, based on which a preliminary set of quality indicators was developed. Second, experts were contacted, and an expert panel was established. Third, the panel members were asked to rate the appropriateness of the quality indicators in a two-round process. Finally, in the fourth step, we evaluated the data by measuring the median and Disagreement Index.

**Results:**

The expert panel consisted of 16 members, congenital cardiologists, nurses, transition experts, patients and research experts. The preliminary set of quality indicators comprised 16 items, categorized in process and structure criteria. Based on the panel’s feedback, the set was refined to 12 quality indicators, which were rated as relevant and feasible.

**Conclusions:**

This study represents the first attempt to develop quality indicators for transitional care services for adolescents with congenital heart disease. The set of 12 quality indicators was developed based on existing evidence and expert opinion. Further testing is needed to assess the feasibility of these quality indicators in daily practice. If successfully implemented, these quality indicators could allow comparison and facilitate benchmarking of transitional care services for adolescents with congenital heart disease.

**Supplementary Information:**

The online version contains supplementary material available at 10.1186/s12913-023-10183-6.

## Background

 Over the past half century, significant improvements have been made in the surgical, interventional, and non-interventional treatment of congenital heart disease (CHD) worldwide [1, 2]. These developments have resulted in improved outcomes for patients, allowing more children born with CHD to reach adulthood. In high-income countries, depending on the region and hospital situation, over 90% of CHD patients reach adulthood [1, 3]. Despite these improvements, CHD patients can often not be considered cured, due to potential late complications such as arrhythmia, heart failure, re-operations, and pregnancy [4]. Therefore, the latest guidelines on the organisation of care recommend the transfer of care to an adult congenital heart disease (ACHD) centre [5], where an ACHD specialised healthcare team takes care of this growing patient group. Transition programmes have been recommended to support adolescents in their transition to adulthood, including the transfer of care to adult healthcare services [6, 7].

According to Blum et al. [8], transition is a complex and multidimensional process that *“attends to the medical, psychosocial and educational/vocational needs of adolescents as they move from child to adult care*”. The aim of transition programmes is to equip adolescents with CHD and their caregivers with essential life skills and help them to navigate the healthcare system [9]. However, evaluating the components of these transition interventions and their potential outcomes can be challenging since numerous interventions are often combined, making it difficult to attribute a specific effect to a particular intervention [10]. In a previous study, we were able to demonstrate that there is a significant variation in transition programmes offered to adolescent CHD patients in European ACHD centres [11].

In recent years, there has been an increasing emphasis on measuring structure and process components for policymakers, healthcare providers, patients, and their caregivers to facilitate comparisons and improvements in the quality of healthcare services. This approach also assists patients and their caregivers in making informed decisions about their care [12]. Quality of healthcare can be defined as “*the degree to which health services for individuals and populations are effective, safe and people-centred*” [13]. The development of quality indicators (QIs) has been recommended to improve the quality of healthcare by enabling benchmarking between healthcare services and facilitating quality measurements [14, 15]. QIs can be classified in structure, process and outcome indicators and include numerators and denominators to facilitate comparison and to measure trends over time [16].

The importance of implementing transition programmes has been recognized, and key elements of generic transition programmes have been published [17–19]. A recent review identified education, continuity of care, satisfaction and self-management as common QIs themes [20]. In the field of CHD, Mackie et al. (2022) showed the benefit of a 1-h nurse led transition intervention on transition readiness and CHD knowledge in adolescents aged 13 to 14 years [21]. Meanwhile, data from the Stepstones project, has been published [22]. The RCT project was effective in implementing a person-centred transition programme for adolescents with CHD aged 16–18.5 years. The programme included eight transition components (i) a transition coordinator (ii) provision of information, (iii) meeting with peers, (iv) written transition plan, (v) availability by phone, e-mail and text-messages, (vi) guidance of parents, and (vii) transfer to adult care [23]. First results revealed a positive effect on adolescents’ empowerment, continuity, confidentiality and talking about sensitive issues was crucial to them. Parents saw their role as contradictory, some expressing the need for more guidance, while others did not. A transition coordinator, trained in adolescent health, played an important role in this process [24].

As demonstrated in our previous work in the field of congenital heart disease, the structure and processes required to identify potential outcomes vary between European ACHD centres [11]. To overcome this gap, QIs based on an evidence based framework for transition programmes for adolescents with congenital heart disease are warranted. Delphi techniques have shown to be beneficial if evidence is lacking or insufficient for the development of QIs [25]. Thus, the objective of the present study is to develop a set of evidence-based QIs for the dimensions of transitional care in congenital heart disease, based on the literature. These QIs should be useful for European centres caring for adolescents with CHD during their transition to adult care. Additionally, the study aims to evaluate these QIs using the opinions of professional experts and CHD patients.

## Methods

**Fig. 1:**
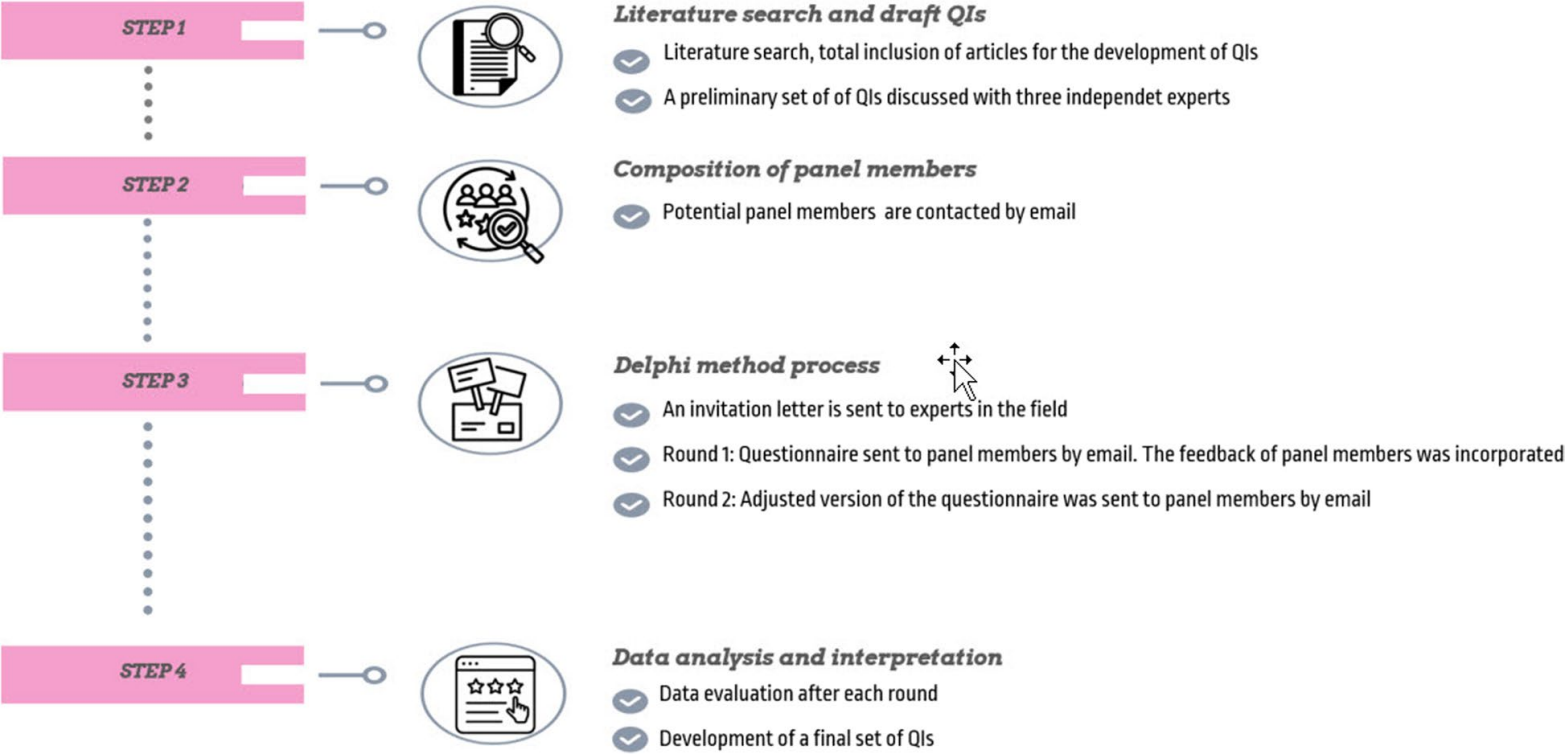
RAND®/University of California at Los Angeles (UCLA) Appropriateness modified Delphi panel process

We used the RAND®/University of California at Los Angeles (UCLA) Appropriateness modified Delphi panel technique to advance QIs. This widely recognized approach combines up-to-date scientific evidence with the expertise of an expert panel to reach agreement on the appropriateness of healthcare practices. During this online process, the expert panel evaluates the scientific findings and generates a statement of appropriateness [26]. Following the method illustrated in Fig. 1, a literature search was conducted, a set of QIs was drafted and then evaluated by an expert panel in two rounds. The process allowed panel members to suggest adding and deleting QIs, making adjustments. Panel members’ suggestions are collected and presented to the group in the next round. For reporting, we used the revised Standards for Quality Improvement Reporting Excellence (SQUIRE 2.0) [27]. According to the Institutional Review Board of Leuven, Belgium, ethical review was not necessary since the study was considered as a service evaluation.

### Data collection

#### Step 1: literature search and draft QIs

A scoping review was performed on CHD adolescent patients aged 10–25 years with focus on transitional care structure and process measures. As a basis, we used the data from a former scoping review on the amount of literature on transfer and transition in young persons with chronic conditions. The authors used MEDLINE, CINAHL, Scopus, and Web of Science databases for the search of relevant publications. The search included quantitative and qualitative study design or mixed methods. This scoping review included 952 studies [28, 29]. We expanded the search string from January 2016 until January 2021 with a focus on congenital heart disease. Another additional 33 studies were added, now evolving into a total of 985 articles.

**Fig. 2:**
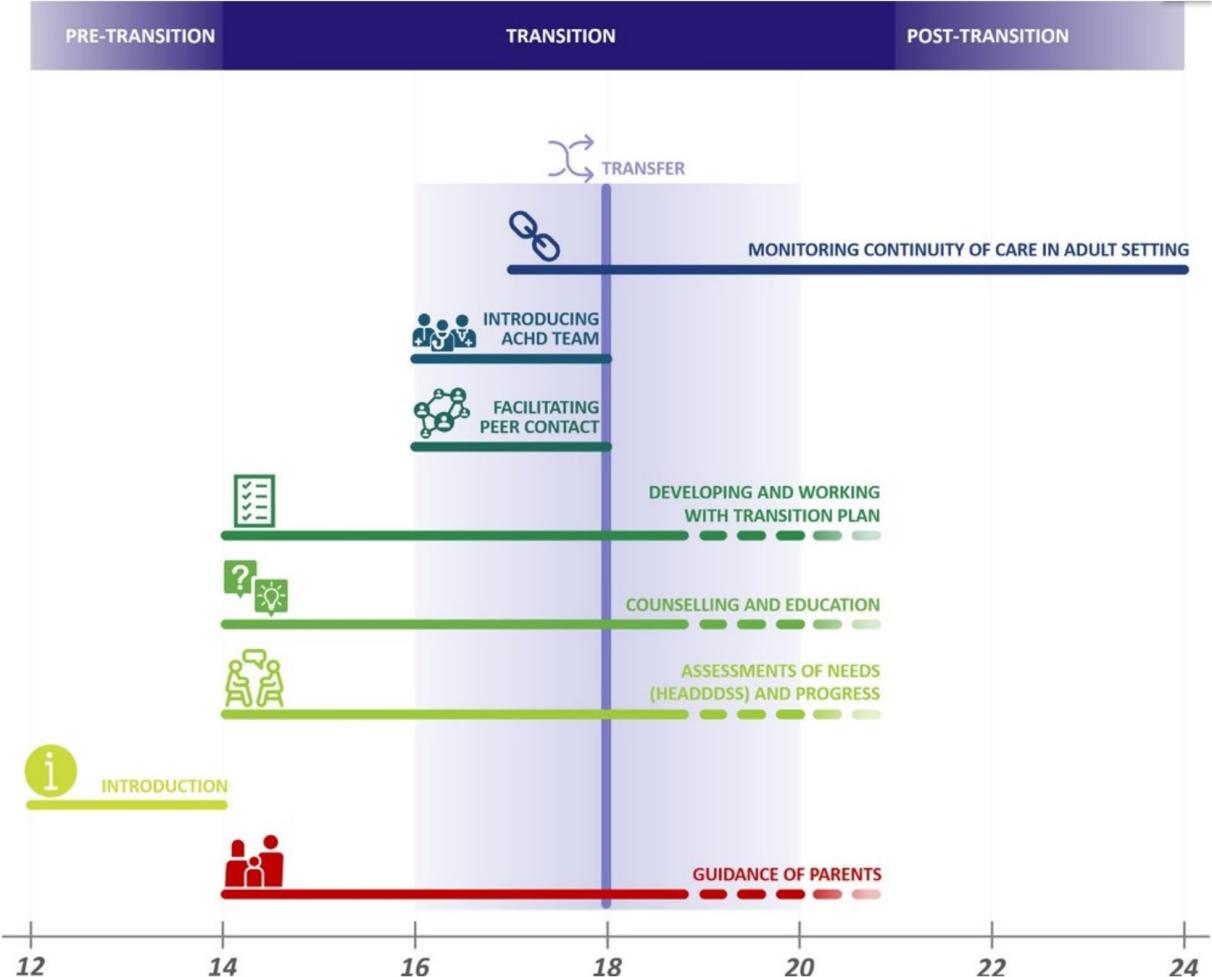
Transition framework. (Reproduced from Moons P, Bratt EL, De Backer J, Goossens E, Hornung T, Tutarel O, et al. Transition to adulthood and transfer to adult care of adolescents with congenital heart disease: a global consensus statement of the ESC Association of Cardiovascular Nursing and Allied Professions (ACNAP), the ESC Working Group on Adult Congenital Heart Disease (WG ACHD), the Association for European Paediatric and Congenital Cardiology (AEPC), the Pan-African Society of Cardiology (PASCAR), the Asia-Pacific Pediatric Cardiac Society (APPCS), the Inter-American Society of Cardiology (IASC), the Cardiac Society of Australia and New Zealand (CSANZ), the International Society for Adult Congenital Heart Disease (ISACHD), the World Heart Federation (WHF), the European Congenital Heart Disease Organisation (ECHDO), and the Global Alliance for Rheumatic and Congenital Hearts (Global ARCH). Eur Heart J. 2021;42(41):4213-23 published under the CC BY-NC license)

Articles were included if they addressed (i) congenital heart disease, (ii) adolescents or young adults with CHD, (iii) parents of adolescents with CHD, and (iv) healthcare providers’ views and experiences with transfer and/or transition. In a second step, the articles had to address the components related to transfer and/or transition of care, as defined by Moons et al. [9], see Fig. 2. This includes (i) Monitoring continuity of care in an adult setting, (ii) Introducing ACHD team, (iii) Facilitating peer contact, (iv) Developing and working with transition plan, (v) Counselling and education, (vi) Assessments of needs and progress, (vii) Introduction, (viii) Guidance of parents.

**Fig. 3:**
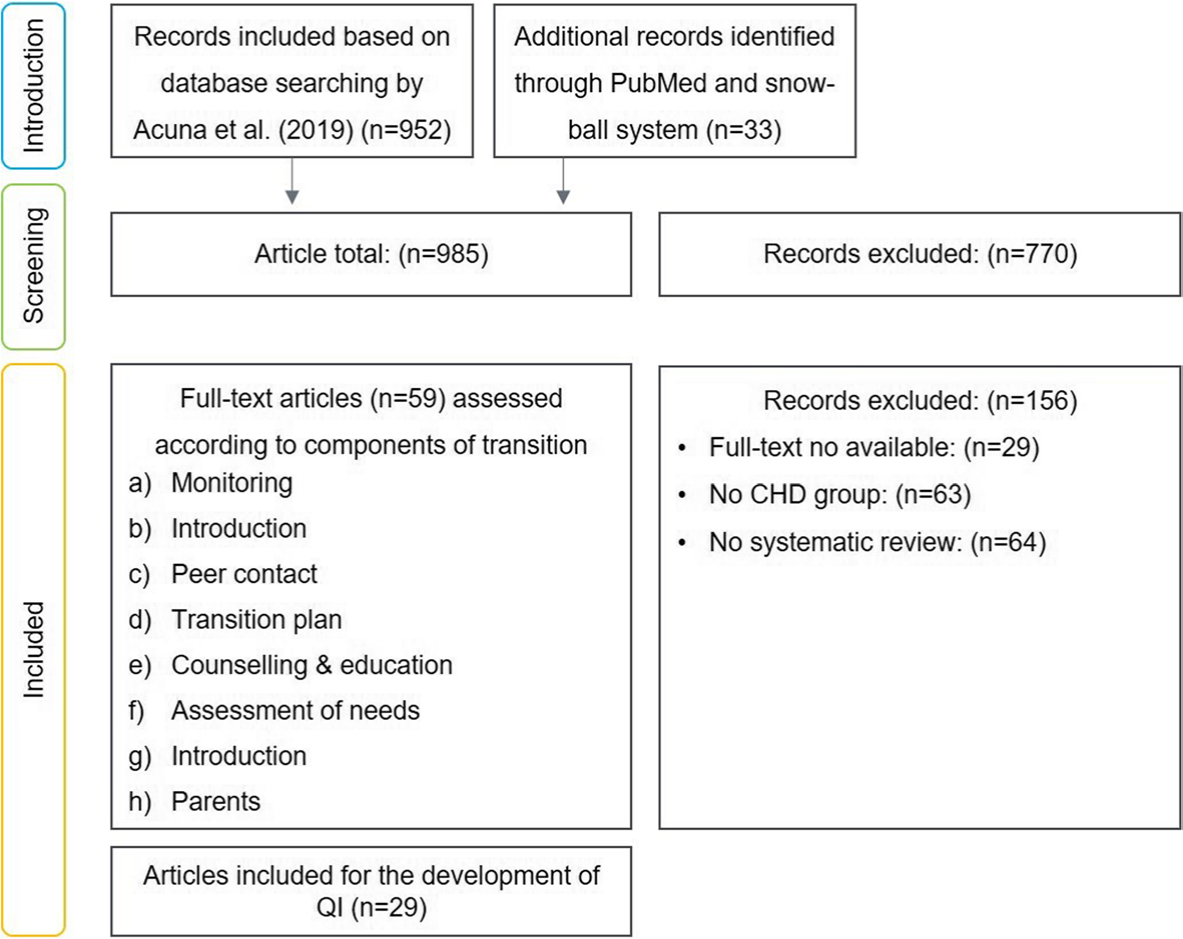
Flowchart of the search process

Titles and Abstracts were screened by the first author (CT), this yielded 29 studies, see Fig. 3.

Based on the findings, QIs were developed using the numerator and denominator approach. The numerator describes the target population that has met the indicator, while the denominator defines the target population that is eligible for the measurement. For each quality indicator, there is a detailed description and explanation of how it can be applied in daily care, including the corresponding level of evidence and the recommended frequency of rating.

The articles included were grouped into qualitative, quantitative or mixed methods studies. Levels of evidence were categorized according to the New Joanna Briggs Institute’s Level of Evidence [30]. The research methods were categorized from Level 1: systematic review to Level 5: expert opinion.

We adhered to the recommended guidelines for using and reporting the Delphi method [25] to ensure content validity and reliability. Following the development of a preliminary set of QIs, three independent experts in the field reviewed the QIs for (i) any relevant indicators that were omitted, (ii) over-representation of indicators, and (iii) inclusion of irrelevant indicators [31].

#### Step 2: composition of panel members

An expert panel was defined as “a *group of knowledgeable people; those who can provide relevant input to the process, have the highest authority possible and are committed and interested*” [32]. Eligible members included paediatric and ACHD cardiologists, nurses, patient representatives, and researchers working on transition. For this study, a combination of convenience and purposive sampling was used to identify experts in the field of CHD, who could provide input on relevant and feasible QIs in transitional care. Potential panel members were contacted via email if they met one or more of the following criteria, (i) had published in the field of CHD, (ii) had a transition programme in their clinic or (iii) were patients with direct or indirect experience with transition. Based on our network, we identified 24 experts, with a focus on Europe, but also included international experts to complete the panel.

#### Step 3: Delphi method process

An invitation letter was sent to 24 transition experts by email, asking them to participate in the study. Written informed consent was obtained from all participants included in the study. With a response rate of 66.6%, 16 experts participated. The questionnaire, which included the QIs, was sent by email and included sociodemographic information such as: (i) type of hospital, (ii) age of participant, and (iii) sex. Panel members were asked to rate the feasibility and relevance of each quality indicator on a scale from 1 to 9 (1 = highly not relevant/not feasible; 9 = highly relevant/feasible). Relevance was defined as: “*The content of the respective QI is relevant for high quality care for patients with congenital heart disease in the transition to adult care*”. An indicator is considered feasible, if valid, reliable and consistent information can be collected, “*either from the medical record or through patient or proxy surveys or interviews and likely to be accurate*” [33].

Panel members were invited to provide feedback on each QI and suggest any necessary adaptations. Those who completed round 1, were included in round 2, where they received an adjusted version of the questionnaire based on the feedback from round 1. The panel was once again asked to rate the relevance of the QI on a scale of 1 to 9. Each panel member was able to view their previous rating, the median rating of the panel, and the disagreement index for each QI. Panellists were asked to consider their ratings from round 1 and adjust them accordingly based on the changes made –see Supplementary Table 1, Additional file 1 for an example.

Reminder emails were sent 2 weeks following the initial email if no answer was received. A second reminder email was sent after the initially proposed deadline.

#### Step 4: data analysis and interpretation

After each round, ratings and comments were summarised. Based on the RAND/UCLA method, all QIs for which a median panel rating between 7 and 9 was reached without disagreement, were rated as appropriate. Median panel ratings between 4 and 6 or any median with disagreement were labelled as uncertain. A median panel rating between 1 and 3 without disagreement was indicated as inappropriate. To be included in the final set, indicators had to have a median panel rating > 7 and no disagreement among panel members’ rating. To detect dispersion between panel ratings, the level of disagreement was assessed by calculating the Disagreement Index (DI) [26]. A disagreement index < 1 indicates no disagreement. Supplementary Table 2, Additional file 2 presents the formulas used to assess relevance and feasibility. Where a consensus was reached with a median panel score between 7 and 9 and no disagreement, the indicator was clear and needed little or no modifications. If the QI was labelled as uncertain, further adaptations were made to increase clarity. Analysis were conducted using Excel 2016.

## Results

The panel included four patient representatives, three paediatric cardiologists, three ACHD cardiologist, three CHD transition experts and three research experts in transition. Table 1 contains information on the characteristics of the panel members. The participants represented various countries in Europe and the USA, including Austria, Belgium, France, Ireland, Switzerland, Sweden, the United Kingdom and the USA.

**Table 1:** Characteristics of panel members

Characteristics	n (%)
Background	
Patient	6 (37.5)
Researcher	7 (43.75)
Paediatric cardiologist	3 (18.75)
ACHD cardiologist	5 (31.25)
Other	1 (6.25)
Working place	
Academic hospital	10 (62.5)
University	1 (6.25)
Other	5 (31.25)
Age	
20–29	1 (6.25)
30–39	3 (18.75)
40–49	5 (31.25)
50–59	5 (31.25)
60–69	2 (12.5)
Sex	
Female	10 (62.5)
Male	6 (37.5)

**Fig. 4:**
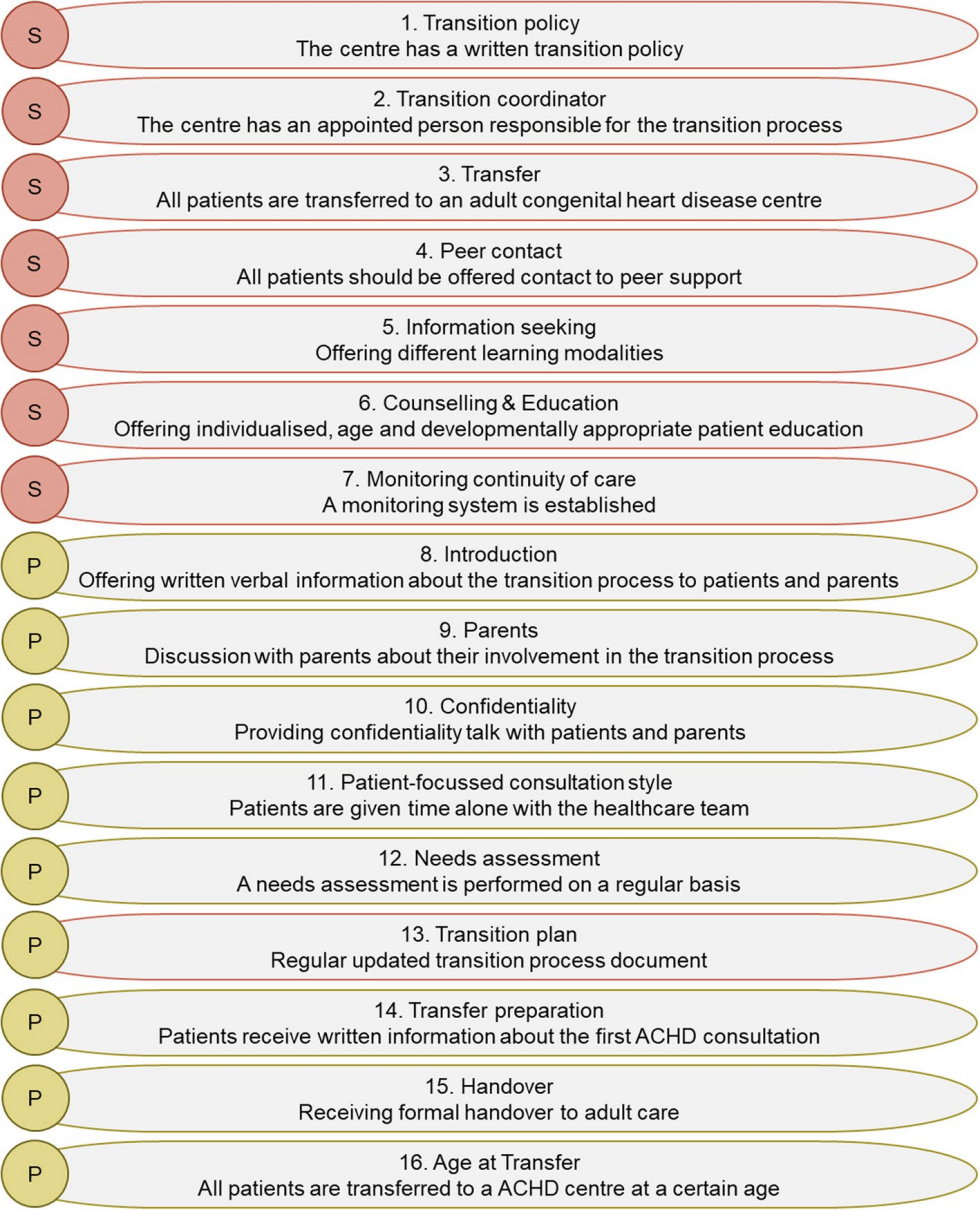
Initial set of quality indicators send to panel members. S= Structure, P= Process

Each QI was accompanied by a definition and explanations of its use in the studies. Based on this process, 15 QIs were developed from the literature review. After presenting these QIs to three independent experts with research and practical experience in transition, one additional QI was added, resulting in an initial set of 16 QIs. These QIs were then categorized into seven structure and nine process criteria (Fig. 4). Supplementary Table 3, Additional file 3, contains detailed information on the initial set of QIs.

In round 1, we achieved a response rate of 100% (*n* = 16). Sixteen QIs were assessed, and they received a median score ≥7 for relevance and no disagreement (DI <1).

**Fig. 5:**
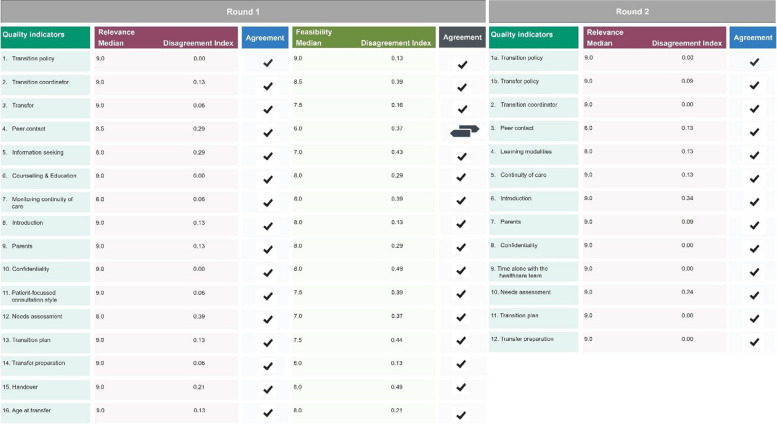
Results of QI rating after Round 1 and Round 2. ✔= appropriate,  = uncertain

The QI “4. Peer contact” was rated as relevant (median ≥7, DI <1) but had uncertain feasibility (median ≤ 6.0, DI < 1), as presented in Fig. 5.

**Fig. 6:**
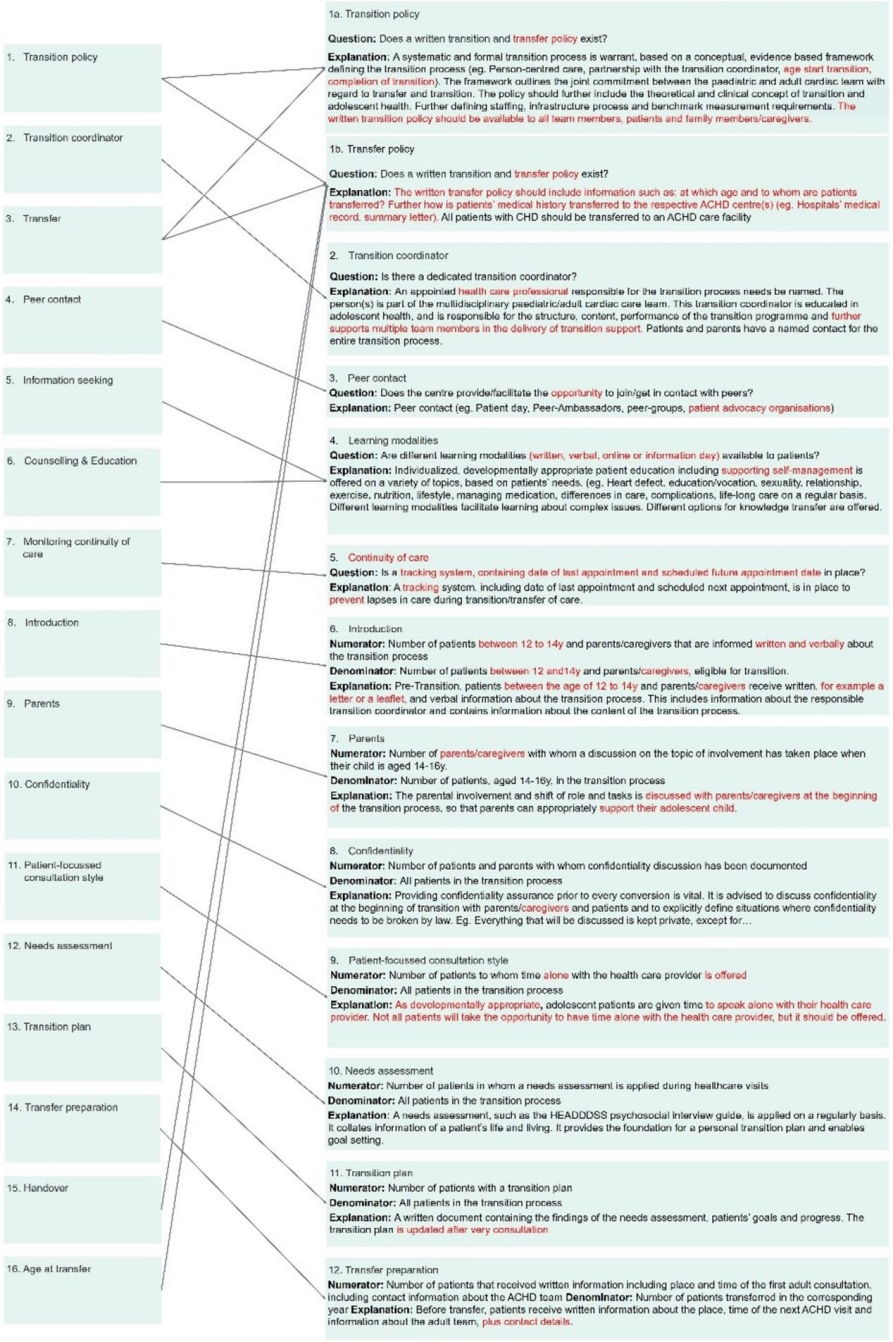
Merged and shifted quality indicators, indicated by the arrow, including semantic changes, indicated as red

The panel recommended adjustments for 15 of the 16 QIs. Semantic adjustments were implemented for 10 of the 16 QIs. Similar comments were grouped and modifications were made accordingly. Following the feedback received for QIs “1. Transition policy” and “3. Transfer” they were merged to form the “Transition and Transfer policy” QIs 1a and 1b, respectively. Furthermore, since panel members indicated significant overlap between QIs “15. Handover” and “16. Age at transfer”, they were combined with the Transition and Transfer policy QIs 1a and 1b. Overlap was also identified between QIs “5. Information seeking” and “6. Counselling & Education”, leading to the creation of a new QI called “Learning Modalities”. As a result, Round 1, yielded 12 QIs, comprising five structure and seven process criteria, see Fig. 6.

The second round elicited a response rate of 87.5% (*n* = 14). However, despite sending three reminder emails, two panel members did not provide a response in the second round.

The relevance of all QIs was rated with a median score of 7–9 and a DI < 1, see Fig. 5. Consequently, 12 QIs (75%) were selected for the final set. For a comprehensive list of the final set of QIs, which includes a definition, numerator, denominator, explanation, corresponding level of evidence, and recommended frequency for rating each QI, refer to Supplementary Table 4, Additional file 4.

## Discussion

The aim of this study was to develop a set of evidence-based QIs for comparability of transition programmes offered to CHD patients in Europe. Using a modified online Delphi technique in two rounds, experts proposed 12 QIs for adolescents with CHD during their transition to adult care. The panel members did not suggest any additional QIs. Instead, overlap in the explanation of the QIs led to the merging of the initial 16 QIs into the final set of 12 QIs after the first round of review. Eight of the 12 QIs align with the components of the transitional care framework (Fig. 2), allowing for their better measurement. Among the four remaining QIs, the “transfer and transition policy” underscores the significance of having a written policy in place to ensure a structured and formal transition process. During the panel discussion the panel members emphasized the importance of ensuring that the transition policy is accessible to all team members, patients, and caregivers. A clear and understandable policy could facilitate the implementation of the transitional care process in daily care delivery. This approach helps patients and caregivers view the transitional care process as an integral part of the care pathway, promoting better understanding and acceptance of the transition policy.

The QI “transition coordinator” advocates for a designated healthcare professional responsible for managing and overseeing the transitional care process. The role of a professional responsible for overseeing the transitional care process has been suggested as a key element of successful transition process [24]. However, the implementation of a transition coordinator model may prove challenging due to financial resource constraints [9, 32]. While the need for a transition coordinator was not discussed in this study, it is noteworthy that the panel members were from high-income countries where such constraints may have been less relevant. It is therefore important to recognize the potential difficulties associated with implementing a transition coordinator model, particularly in low-income settings where financial resources may be more limited.

The acceptance of the two QIs, “Confidentiality” and “Time alone with the healthcare team” indicates that the panel members valued the essence of adolescent health. Discussing confidentiality and providing adolescents with private time with the healthcare team has proven beneficial, as they are more likely to open up about sensitive topics such as sexuality, mental health issues, or suicidal thoughts [34].

In a recent study by Bailey et al. (2022) a systemic review was conducted regarding QIs for the transition to adult care. They found nine studies that published QIs related to the transition from paediatric to adult care, with a primary focus on patient-centred outcomes and a lesser emphasis on developmental and psychological aspects [20]. However, since all of those aspects significantly influence adolescents’ well-being during the transition to adult care, there is a clear need for a comprehensive transition programme.

Such a programme should encompass essential components of transition, regardless of the personal or financial resources available at CHD centres. It is crucial to understand the content of individual transition practices and analyse them, as there is little impact on the overall quality of care offered without this knowledge [32]. The set of QIs allows the measurement and comparison of different transition programmes, while still offering opportunities for patient and organisational flexibility. With this in mind, we have refrained from proposing a specific age for transfer, for instance.

Achieving improvement in quality requires a comprehensive understanding of the interconnectedness between structure, process, and outcome. Although outcomes are often seen as the gold standard for assessing quality, drawing valid conclusions can be difficult due to the influence of various factors on the outcome [35]. Since the transitional care process is a complex intervention with multiple factors influencing outcomes, we decided to focus on process and structure indicators rather than on outcome criteria in our analysis. Specifically, we deliberately excluded outcome measures after reviewing the literature to lay the foundation for future research on this topic. Instead, we collected process and structure indicators that are more amenable to measuring the implementation and effectiveness of the transitional care process. By doing so, we aimed to enhance our understanding of the mechanisms and determinants underlying successful transitions of care and to identify areas for improvement in clinical practice.

Our expert panel consisted of 16 members, which aligns with other Delphi panels, generally composed of 15 to 20 panel members [25]. To achieve heterogeneity in our study, we engaged stakeholders from different countries and professional backgrounds. To ensure content validity, we covered all dimensions of transitional care, including patient and caregiver-related aspects, as well as incorporating structural elements to facilitate the process. By employing these strategies, we aimed to enhance the rigor and generalisability of our findings and to ensure that all relevant aspects of the transitional care process were adequately represented.

A common understanding was reached in two rounds through the collaboration of an international and multidisciplinary healthcare and patient panel. This approach fosters a shared understanding within the field of congenital heart disease. Considering potential time constraints for the panel members, we purposefully opted against a moderated online panel discussion after round 1.

However, the results must be considered in the context of certain limitations. We evaluated feasibility in the first round. Panel members expressed concerns about the feasibility of “peer support”, “learning modalities” and “confidentiality”, indicating that these measures may be difficult to collect easily through medical records. Before the set of QIs can be used in daily practice, feasibility needs to be tested. This research is currently in progress. Furthermore, we did not include caregivers in our expert panel, although we are aware of their importance in the transitional care process. The role of caregivers in the transitional care process is one of ambivalence, as they are tasked with caring for the child while also promoting their independence [24, 36]. The role of caregivers is even more important in case of a child with cognitive or learning problems. To address this issue, we included the QI “parents” in our panel to initiate a discussion about their role and shift of care at the beginning of the transitional care process. However, to ensure a more comprehensive and nuanced understanding of the transitional care process, future research should include separate expert panel for patients, caregivers, and healthcare professionals. Attention ought to be given to caregivers of children with learning disabilities. This could help to prevent misinterpretations and provide a more accurate representation of the different perspectives and needs involved in the transitional care process.

To our knowledge, this study is the first of its kind in the field of CHD to enable quality improvement, benchmarking and comparison of transition care services. This has potential implications for policymakers, healthcare providers and patients and their caregiver. Moreover, it underscores the importance of evaluating the effectiveness of these interventions from a research perspective.

## Conclusion

This study is a pioneering effort in the field of congenital heart disease to establish a set of QIs for transitional care services. The set of 12 QIs was developed based on existing evidence. Consensus on their relevance in daily practice was reached through collaboration among multidisciplinary healthcare and patient representatives. For centres planning to implement transitional care services, the QIs can offer guidance on essential components and serve as a foundation for discussion with hospital and governmental policymakers. Further testing is required to assess the feasibility of implementing these QIs in centres offering CHD transitional care services. If successful, incorporating QIs into transition programs could help ensure adolescents receive high-quality care tailored to their unique needs.

### Supplementary Information

**Additional file 1: Supplementary Table 1.** Example of a QI rating scheme in round 2.

**Additional file 2: Supplementary Table 2.** Excel 2016 formulas used to assess relevance and feasibility.

**Additional file 3: Supplementary Table 3.** Initial set of QIs for Round 1.

**Additional file 4: Supplementary Table 4.** Final Set of QIs.

## Data Availability

Final data generated or analysed during this study are included in this published article [and its supplementary information files]. Literature search, Feedback datasets of the panel members used and/or analysed during the current study are available from the corresponding author on reasonable request.

## Abbreviations

ACHD: Adult Congenital Heart Disease
CHD: Congenital Heart Disease
DI: Disagreement Index
QIs: Quality Indicators
RAND®/UCLA: RAND®/University of California at Los Angeles
USA: United States of America
