# Supplementary figures and images for: Development of quality indicators of transfer and transition in adolescents and young adults with congenital heart disease
Source: BMC Health Serv Res. 2023 Oct 25;23:1154. doi: 10.1186/s12913-023-10183-6 (PMC10601126; doi:10.1186/s12913-023-10183-6)

Additional file 1

Supplementary Table 1: Example of a QI rating scheme in round 2


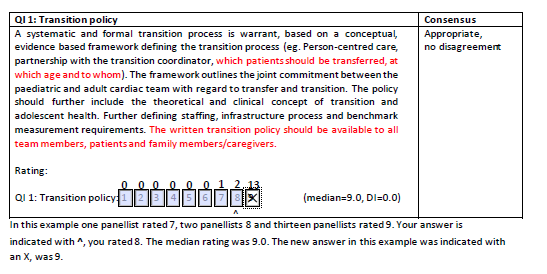

Supplement: Supplementary file 1 — Additional file 1: Supplementary Table 1. Example of a QI rating scheme in round 2. [file 12913_2023_10183_MOESM1_ESM.docx]
