# Supplementary material for: Development of quality indicators of transfer and transition in adolescents and young adults with congenital heart disease
Source: BMC Health Serv Res. 2023 Oct 25;23:1154. doi: 10.1186/s12913-023-10183-6 (PMC10601126; doi:10.1186/s12913-023-10183-6)
Supplement: Supplementary file 2 — Additional file 2: Supplementary Table 2. Excel 2016 formulas used to assess relevance and feasibility. [file 12913_2023_10183_MOESM2_ESM.docx]

Additional file 2

Supplementary Table 2: Excel 2016 formulas used to assess relevance and feasibility

| Median | =MEDIAN(xx;xx) |
| --- | --- |
| 30^th^ percentile | =QUANTIL.INKL(xx:xx;0.3) |
| 70^th^ percentile | =QUANTIL.INKL(xx:xx;0.7) |
| Interpercentile range (IPR), 70^th^-30^th^ | =70^th^ percentile-30^th^ percentile |
| Central point of IPR (IPRCP) | =((70^th^ percentile+30^th^ percentile)/2) |
| Asymmetry Index (AI) | =5-IPRCP |
| Interpercentile Range adjusted for symmetry required for disagreement (IPRAS) | =2.35+(1.5*AI) |
| Disagreement Index | IPR/IPRAS |
