# Supplementary material for: Development of quality indicators of transfer and transition in adolescents and young adults with congenital heart disease
Source: BMC Health Serv Res. 2023 Oct 25;23:1154. doi: 10.1186/s12913-023-10183-6 (PMC10601126; doi:10.1186/s12913-023-10183-6)
Supplement: Supplementary file 3 — Additional file 3: Supplementary Table 3. Initial set of QIs for Round 1. [file 12913_2023_10183_MOESM3_ESM.docx]

Additional file 3

Supplementary Table 3: Initial set of Quality Indicators for Round 1

| Structure | **QI 1: Transition policy**  The centre has a written transition policy  **Question:** Does a written transition policy exist?  **Answer:** yes/no  **Rating:** annual  **Explanation:** A systematic and formal transition process is warrant, based on a conceptual, evidence based framework defining the transition process (eg. Person-centred care, partnership with the transition coordinator). The framework outlines the joint commitment between the paediatric and adult cardiac team with regard to transfer and transition. The policy should further include the theoretical and clinical concept of transition and adolescent health. Further defining staffing, infrastructure process and benchmark measurement requirements.  McLoughlin et al., (2018), Clarizia et al., (2009), Luyckx et al., (2011) |
| --- | --- |
| Structure | **QI 2: Transition coordinator**  The centre has an appointed person responsible for the transition process  **Question:** Has a transition coordinator been appointed for the transition process?  **Answer:** yes/no  **Rating:** annual  **Explanation:** An appointed person, responsible for the transition process needs be named. The person(s) is part of the multidisciplinary paediatric/adult cardiac care team. Educated in adolescent health, responsible for the structure, content and performance of the transition programme. Patients and parents have a named contact for the entire transition process.  Saarijärvi et al., (2021), de Hosson et al., (2021), Thomet et al., (2018), Thomet et al., (2021) |
| Structure | **QI 3: Transfer**  All patients are transferred to an adult congenital heart disease centre  **Question:** Does a defined transfer policy exist with an adult CHD clinic?  **Answer:** yes/no  **Rating:** annual  **Explanation:** All patients with CHD should be transferred to ACHD care.  Baumgartner et al., (2014) |
| Structure | **QI 4: Peer contact**  All patients should be offered contact to peer support  **Indicator:** Structure  **Question:** Is there the possibility to join/get in contact with peers? Answer: yes/no  **Rating:** annual  **Explanation:** Peer contact (eg. Patient day, Peer-Ambassadors, peer-groups)  Saarijärvi et al., (2021), Lopez et al., (2015) |
| Structure | **QI 5: Information seeking**  Offering different learning modalities  **Question:** Are different learning modalities available to patients?  **Answer:** yes/no  **Rating:** annual  **Explanation:** Different learning modalities facilitate learning about complex issues. Different options for knowledge transfer are offered.  Saarijärvi et al., (2021), Lopez et al., (2015), Lopez et al., (2018), Rempel et al., (2014) |
| Structure | **QI 6: Counselling & Education**  Offering individualised, age and developmentally appropriate patient education  **Question:** Is individualized, age and developmentally appropriate patient education offered to the patients?  **Answer:** yes/no  **Rating:** annual  **Explanation:** Individualized, developmentally appropriate patient education is offered on a variety of topics, based on patients’ needs. (eg. Heart defect, education/vocation, sexuality, relationship, exercise, nutrition, lifestyle, managing medication, differences in care, complications, life-long care on a regular basis.  Lopez et al., (2015), Mackie et al., (2014), Uzark et al., (2015), Mackie et al., (2018), Bratt et al., (2015), Clarizia et al., (2009) |
| Structure | **QI 7: Monitoring continuity of care**  A monitoring system is established  **Question:** Is a monitoring system in place?  **Answer:** yes/no  **Rating:** annual  **Explanation:** A monitoring system is in place to prevent loss-to follow up during transition/transfer of care.  Mackie et al., (2019) |
| Process | **QI 8: Introduction**  Offering written or verbal information about the transition process to patients and parents  **Numerator:** Number of patients and parents informed about the transition process.  **Denominator:** Number of patients by the age of 14 years and parents, eligible for transition.  **Rating:** annual  **Explanation:** Pre-Transition, patients and parents receive written and verbal information about the transition process. This includes information about the responsible transition coordinator and contains information about the content of the transition process.  Thomet et al., (2018), Bratt et al., (2015), van Staa et al., (2011) |
| Process | **QI 9: Parents**  Discussion with parents about their involvement in the transition process  **Numerator:** Number of parents with whom a discussion on the topic of involvement has taken place  **Denominator:** Number of patients, age 14-16, in the transition process  **Rating:** annual  **Explanation:** The parental involvement and shift of role and tasks during the transition process is negotiated together with patients and the parents.  McLoughlin et al., (2018), Clarizia et al., (2009), Saarijärvi et al., (2021), van Staa et al., (2011), Bratt et al., (2018), de Hosson et al., (2021), Catena et al., (2018), Burström et al., (2019) |
| Process | **QI 10: Confidentiality**  Providing confidentiality talk with patients and parents  **Numerator:** Number of patients and parents with whom confidentiality discussion has been documented  **Denominator:** All patients in the transition process  **Rating:** annual  **Explanation:** Providing confidentiality assurance prior to every conversion is vital. It is advised to discuss confidentiality at the beginning of transition with parents and patients and to explicitly define situations where confidentiality needs to be broken by law. Eg. Everything that will be discussed is kept private, except for…  Saarijärvi et al., (2021) |
| Process | **QI 11: Patient-focussed consultation style**  Patients is given time alone with the health care team  **Numerator:** Number of patients receiving patient focussed consultation time with the health care provider  **Denominator:** All patients in the transition process  **Rating:** annual  **Explanation:** Patients are given time with the health care team without parents present.  Clarizia et al., (2009), Thomet et al., (2018) |
| Process | **QI 12: Needs assessment**  A needs assessment is performed on a regular basis  **Numerator:** Number of patients a need assessment is applied regularly  **Denominator:** All patients in the transition process  **Rating:** annual  **Explanation:** A needs assessment, such as the HEADDDSS psychosocial interview guide, is applied on a regularly basis. It collates information of a patient’s life and living. Provides the foundation for a personal transition plan and enables goal setting.  Saarijärvi et al., (2021), Thomet et al., (2018), Lopez et al., (2015), Deng et al., (2019) |
| Structure | **QI 13: Transition plan**  Regular updated transition process document  **Numerator:** Number of patients with a regularly updated transition plan  **Denominator:** All patients in the transition process  **Rating:** annual  **Explanation:** A written document containing the findings of the needs assessment, patients’ goals and progress.  McLoughlin et al., (2018), Saarijärvi et al., (2021) |
| Process | **QI 14: Transfer preparation**  Patients receive written information about the first ACHD consultation  **Numerator:** Number of patients that received written information including place and time of the first adult consultation, including contact information about the ACHD team  **Denominator:** Number of patients transferred in the corresponding year  Rating: annual  **Explanation:** Before transfer, patients receive written information about the place, time of the next ACHD visit and information about the adult team, including contact details and allowance of parents at the adult clinic.  de Hosson et al., (2021), van Staa et al., (2011), Reid et al., (2004), Asp et al., (2015), Moons et al., (2009), Ochiai et al., (2019) |
| Process | **QI 15: QI 15: Handover**  Receiving a formal handover to adult care  **Numerator:** Number of patients having received transfer communication either with the adult team or the transition coordinator from the adult side  **Denominator:** Number of patients with last visit at the paediatric clinic  **Rating:** annual  **Explanation:** Formal handover. Having joint consultation with the adult team or a transition coordinator serving both sides.  Saarijärvi et al., (2021), Thomet et al., (2018), Burström et al., (2016) |
| Process | **QI 16: Age at Transfer**  All patients are transferred to a ACHD centre at a certain age  **Numerator:** Number of patients 16-20y with their first visit at adult care  **Denominator:** All patients in this age range, not considered to be cured.  **Rating:** annual  **Explanation:** All patients are transferred at latest at age 20, according to patients preferences, physical, emotional maturity or institutional recommendations  McLoughlin et al., (2018), de Hosson et al., (2021), Rutishauser et al., (2014), Gaydos et al., (2020), Rutishauser et al., (2011), Reid et al., (2004) |
