# Supplementary material for: Development of quality indicators of transfer and transition in adolescents and young adults with congenital heart disease
Source: BMC Health Serv Res. 2023 Oct 25;23:1154. doi: 10.1186/s12913-023-10183-6 (PMC10601126; doi:10.1186/s12913-023-10183-6)
Supplement: Supplementary file 4 — Additional file 4: Supplementary Table 4. Final Set of QIs. [file 12913_2023_10183_MOESM4_ESM.docx]

Additional file 4: Supplementary Table 4: Final Set of QI

| **ORGANISATIONAL ASPECTS** | \| **QI 1a&b: Transition and transfer policy**  The centre has a written transition and transfer policy  **QI 1a: Transition policy** \| \| --- \| \| **Indicator:** Structure  **Numerator:** Does a written transition policy exist?  **Denominator:** yes/no  **Rating:** annual  **Explanation:** A systematic and formal transition process is warrant, based on a conceptual, evidence based framework defining the transition process (eg. person-centred care, partnership with the transition coordinator, age start transition, completion of transition). The framework outlines the joint commitment between the paediatric and adult cardiac team with regard to transfer and transition. The policy should further include the theoretical and clinical concept of transition and adolescent health. Further defining staffing, infrastructure process and benchmark measurement requirements. The written transition policy should be available to all team members, patients and family members/caregivers.  **Level of evidence:** 3 & 4 (McLoughlin et al. (2018), Clarizia et al. (2009), Luyckx et al. (2011)  **QI 1b) Transfer policy**  **Indicator:** Structure  **Numerator:** Does a written transfer policy exist?  **Denominator:** yes/no  **Rating:** annual  **Explanation:** The written transfer policy should include information such as: at which age and to whom are patients transferred? Further how is patient’s medical history transferred to the respective ACHD centre(s) (eg. hospital medical record, summary letter). All patients with CHD should be transferred to an ACHD care facility.  **Level of evidence**: Level 4 (Baumgartner et al., (2014), McLoughlin et al., (2018), de Hosson et al., (2021), Rutishauser et al., (2014), Gaydos et al., (2020), Rutishauser et al., (2011), Reid et al., (2004)) \| |
| --- | --- | --- | --- |
|  | **QI 2: Transition coordinator**  The centre has an appointed person responsible for the transition process  **Indicator:** Structure  **Numerator:** Is there a dedicated transition coordinator?  **Denominator:** yes/no  **Rating:** annual  **Explanation:** An appointed health care professional responsible for the transition process needs be named. The person(s) is part of the multidisciplinary paediatric/adult cardiac care team. This transition coordinator is educated in adolescent health, and is responsible for the structure, content, performance of the transition programme and further supports multiple team members in the delivery of transition support. Patients and parents have a named contact for the entire transition process.  **Level of evidence**: Level 4 (Saarijärvi et al., (2021), de Hosson et al., (2021), Thomet et al., (2018), Thomet et al., (2021)) |
|  | **QI 3: Peer contact**  All patients should be offered contact to peer support  **Indicator:** Structure  **Numerator:** Does the centre provide/facilitate the opportunity to join/get in contact with peers?  **Denominator:** yes/no  **Rating:** annual  **Explanation:** Peer contact (eg. patient day, peer ambassadors, peer groups, patient advocacy organisations)  **Level of evidence:** Level 4 (Saarijärvi et al., (2021), Lopez et al., (2015)) |
|  | **QI 4:** **Learning modalities**  Offering different learning modalities and materials  **Indicator**: Structure  **Numerator**: Are different learning modalities and materials (written, verbal, online or information day) available to patients?  **Denominator:** yes/no  **Rating:** annual  **Explanation:** Individualized, developmentally appropriate patient education including support for self-management, is offered on a range of topics, based on patient’s needs. (e.g. heart defect, education/vocation, sexuality, relationships, exercise, nutrition, lifestyle, medication management, differences in care, complications, lifelong care on a regular basis. Different learning modalities and materials facilitate learning about complex issues. Different ways of transferring knowledge are offered.  **Level of evidence**: Level 1 (Saarijärvi et al., (2021), Lopez et al., (2015), Lopez et al., (2018), Rempel et al., (2014) Lopez et al., (2015), Mackie et al., (2014), Uzark et al., (2015), Mackie et al., (2018), Bratt et al., (2015), Clarizia et al., (2009)) |
|  | **QI 5: Continuity of care**  A tracking system is established  **Indicator**: Structure  **Numerator**: Is a tracking system, containing date of last appointment and scheduled future appointment date in place?  **Denominator:** yes/no  **Rating:** annual  **Explanation:** A tracking system, including date of last appointment and scheduled next appointment, is in place to prevent lapses in care during transition/transfer of care.  **Level of evidence:** Level 5 (Mackie et al., (2019)) |
| **PRE-TRANSITION** | **QI 6: Introduction**  Offering written or verbal information about the transition process to patients and parents  **Indicator**: Process  **Numerator**: Number of patients between 12 to 14y and parents/caregivers that are informed written and verbally about the transition process  **Denominator:** Number of patients between 12 and14y and parents/caregivers, eligible for transition.  **Rating:** annual  **Explanation:** Pre-Transition, patients between the age of 12 to 14y and parents/caregivers receive written, for example a letter or a leaflet, and verbal information about the transition process. This includes information about the responsible transition coordinator and contains information about the content of the transition process.  **Level of evidence:** Level 4 (Thomet et al., (2018), Bratt et al., (2015), van Staa et al., (2011)) |
| T**RANSITION** | **QI 7:Parents**  Discussion with parents about their involvement in the transition process  **Indicator**: Process  **Numerator**: Number of parents/caregivers with whom a discussion on the topic of involvement has taken place when their child is aged 14-16y.  **Denominator:** Number of patients, aged 14-16y, in the transition process  **Rating:** annual  **Explanation:** The parental involvement and shift of role and tasks is discussed with parents/caregivers at the beginning of the transition process, so that parents can appropriately support their adolescent child. Parents are supported in their shift or roles, according to their needs.  **Level of evidence:** Level 4 (McLoughlin et al., (2018), Clarizia et al., (2009), Saarijärvi et al., (2021), van Staa et al., (2011), Bratt et al., (2018), de Hosson et al., (2021), Catena et al., (2018), Burström et al., (2019)) |
|  | **QI 8:Confidentiality**  Providing confidentiality talk with patients and parents  **Indicator**: Process  **Numerator**: Number of patients and parents with whom confidentiality discussion has been documented  **Denominator:** All patients in the transition process  **Rating:** annual  **Explanation:** Providing confidentiality assurance prior to every conversion is vital. It is advised to discuss confidentiality at the beginning of transition with parents/caregivers and patients and to explicitly define situations where confidentiality needs to be broken by law. Eg. Everything that will be discussed is kept private, except for…  **Level of evidence:** Level 4 (Saarijärvi et al., (2021)) |
|  | **QI 9:Time alone with the healthcare team**  Patients is given time alone with the health care team  **Indicator:** Process  **Numerator:** Number of patients to whom time alone with the health care provider is offered  **Denominator:** All patients in the transition process  **Rating:** annual  **Explanation:** As developmentally appropriate, adolescent patients are given time to speak alone with their health care provider. Not all patients will take the opportunity to have time alone with the health care provider, but it should be offered.  **Level of evidence:** Level 4 (Clarizia et al., (2009), Thomet et al. ,(2018)) |
|  | **QI 10:Needs assessment**  A needs assessment is performed on a regular basis  **Indicator:** Process  **Numerator:** Number of patients in whom a needs assessment is applied during healthcare visits  **Denominator:** All patients in the transition process  **Rating:** annual  **Explanation:** A needs assessment, such as the HEADDDSS psychosocial interview guide, is applied on a regularly basis. It collates information of a patient’s life and living. As such, it includes their limitations but also their capacities and resources. This assessment provides the foundation for a personal transition plan and enables goal setting.  **Level of evidence:** Level 4 (Saarijärvi et al., (2021), Thomet et al., (2018), Lopez et al., (2015), Deng et al., (2019)) |
|  | **QI 11:Transition plan**  Regular updated transition process document  **Indicator:** Process  **Numerator:** Number of patients with a transition plan  **Denominator:** All patients in the transition process  **Rating:** annual  **Explanation:** A written document containing the findings of the needs assessment, patients’ goals and progress. The transition plan is updated after every consultation  **Level of evidence:** Level 4 (McLoughlin et al., (2018), Saarijärvi et al., (2021)) |
|  |  |
| **POST-TRANSITION** | **QI 12:Transfer preparation**  Patients receive written information about the first ACHD consultation  **Indicator:** Process  **Numerator:** Number of patients that received written information including place and time of the first adult consultation, including contact information about the ACHD team  **Denominator:** Number of patients transferred in the corresponding year  **Rating:** annual  **Explanation:** Before transfer, patients receive written information about the place, time of the next ACHD visit and information about the adult team, plus contact details  **Level of evidence:** Level 4 (de Hosson et al., (2021), van Staa et al., (2011), Reid et al., (2004), Asp et al., (2015), Moons et al., (2009), Ochiai et al., (2019), Saarijärvi et al., (2021), Thomet et al., (2018), Burström et al., (2016)) |

Colours: According to Moons et al. 2021. Transition to adulthood and transfer to adult care of adolescents with congenital heart disease. Eur Heart J. https://doi.org/10.1093/eurheartj/ehab388

Level of Evidence: Based on the levels of evidence by the Joanna Briggs Institute
